# Study on the Micro-Surfacing Properties of SBR Modified Asphalt Emulsion with Reclaimed Asphalt Pavement

**DOI:** 10.3390/ma18040802

**Published:** 2025-02-12

**Authors:** Di Yu, Junchi Luo, Huan Lang, Fang Hua, Yongyong Yang, Meng Xie

**Affiliations:** 1 School of Civil Engineering and Architecture, East China Jiaotong University, Nanchang 330013, China; 2 Jiangxi Communications Investment Group Haitong Highway Maintenance Co., Ltd., Nanchang 330108, China

**Keywords:** micro-surfacing, reclaimed asphalt pavement, SBR emulsion asphalt

## Abstract

With the updates and differences in the usage of reclaimed asphalt pavement (RAP) separation technology, the production of fine-particle RAP exceeds their usage, resulting in an excess of fine-particle RAP. How to apply this excess RAP on a large scale in micro-surfacing technology has become a challenge. This study aims to investigate the advantages and disadvantages of incorporating RAP into micro-surfacing. To this end, a mix design process for RAP-containing micro-surfacing, based on the current gradation design procedure and existing research findings, is proposed. The study examines the influence of six different RAP contents, as well as the effects of SBR emulsified asphalt, added water, and RAP on the micro-surfacing mix design. Subsequently, the effects of RAP content on the pavement performance of micro-surfacing are evaluated through rutting deformation rate, wet wheel abrasion, and British pendulum tests. Finally, an economic analysis from a construction perspective is conducted. The results indicate that the optimized mix design process meets specific usage requirements and is effective for RAP-containing micro-surfacing. The mix design results show that the addition of RAP reduces the asphalt demand and mixing time of slurry mixtures. Increasing the amount of added water can meet mixing requirements, but it leads to a reduction in early strength. As the RAP content increases, skid resistance improves, with a maximum increase of 14.9%; the rutting deformation rate increases, and this is the main factor limiting the RAP content, restricting it to no more than 40%; water damage resistance shows an initial increase followed by a decrease, but this does not affect the RAP content. Therefore, the maximum RAP content is limited to 40% without the addition of other additives, mainly due to the phenomenon of weak agglomeration in RAP. Finally, cost calculations show that incorporating 40% RAP can save approximately 17% of the construction costs.

## 1. Introduction

**Figure 1:**
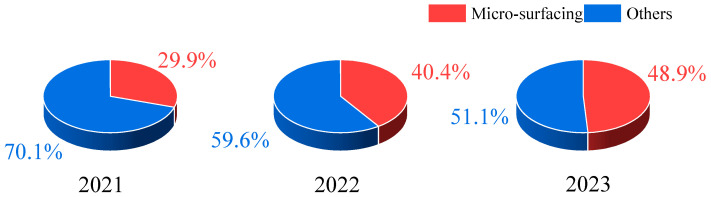
Percentage of micro-surfacing in Jiangxi Province.

Micro-surfacing is a commonly used preventive maintenance technique [1,2]. Since the 1980s, it has been widely adopted in regions such as the United States, Europe, and Canada, as it is an economical, efficient, and environmentally friendly treatment method [3]. China began applying micro-surfacing technology in 1990, and its use has significantly increased over the past decade. According to a survey by Jiangxi Transport Investment Group, Figure 1 shows the construction proportion of preventive maintenance on highways in Jiangxi Province over the past three years. From the figure, it can be seen that micro-surfacing has become increasingly important in highway preventive maintenance.

Each year, a large amount of solid waste is generated from pavement construction and maintenance, which is typically transported back to bases for sorting and reuse [4]. The recycling rate of reclaimed asphalt pavement (RAP) in China is only 30%, much lower than in other countries, where the recycling rate reaches over 80%, such as in Japan and Germany [5]. The reason is that RAP is usually mechanically separated using equipment like roll crushers, impact crushers, or jaw crushers, which can weaken the strength of the aggregates [6]. Worldwide, RAP has been widely applied in hot mix asphalt, while in China, there is a greater preference for using the coarse fraction of RAP in hot recycling, leading to a lower consumption of fine-grained RAP than its production [7,8]. Furthermore, the use of more advanced refined decomposition techniques increases the proportion of fine particles, exacerbating the aforementioned issue [9].

In recent years, researchers have attempted to incorporate solid waste into micro-surfacing technology, facing challenges such as disorganized design processes and reduced mixture performance [10,11,12]. Shabani et al. [13,14] suggested that using industrial waste materials with rough surfaces as a filler in micro-surfacing pavements can effectively increase the cohesion, wear resistance, and deformation resistance of the mixture. Cheng et al. [15,16] used waste glass slag to replace aggregates in micro-surfacing. Although this increased the skid resistance, the glass, being more brittle and less adhesive compared to basalt, reduced the wear resistance and deformation resistance of the micro-surfacing pavement. Wang [11,17] studied the use of RAP in micro-surfacing and found that adding RAP reduced the optimum asphalt-aggregate ratio, shortened mixing time, and improved skid resistance and water damage resistance. However, there is a maximum amount of RAP that can be added, and the use of rejuvenators can alleviate some of these issues. Robati [18] researched the addition of RAP and reclaimed asphalt shingles (RAS) to micro-surfacing. The performance test results indicated that the addition of RAP and RAS significantly reduced cohesion and deformation resistance, but had little impact on water sensitivity. The performance shortcomings limited the amount of RAP and RAS that could be added.

Currently, the design process for micro-surfacing containing RAP still primarily follows the existing ISSA micro-surfacing mix design guidelines, which do not address the design process for mixtures containing RAP [19]. Robati [20] studied the effects of asphalt emulsified, water, and cement on the mix design and found that the results derived from the current design process vary significantly, making it impossible to provide accurate mix design recommendations. Some studies suggest that RAP in micro-surfacing should not be regarded as “black rock” but should consider the fusion of new and old asphalt, which is not addressed in the current guidelines [11,21]. The existing micro-surfacing design guidelines often lead to multiple iterations, increasing both time and material costs. The inclusion of RAP changes the original balance, altering the impact of factors such as aggregate, water, and asphalt content on the micro-surfacing mix design.

**Figure 2:**
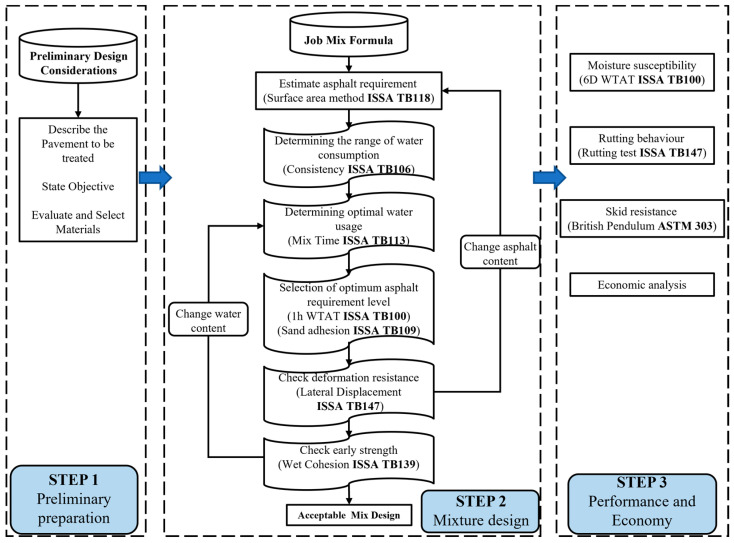
Research flow chart.

To this end, this study aims to determine the maximum allowable RAP content in micro-surfacing through the improvement of the mix design and performance testing of micro-surfacing. The economic advantages of this technology are also considered from a construction perspective. The research framework is shown in Figure 2, and the research objectives are summarized as follows:

- Propose an appropriate mix design for micro-surfacing containing RAP.
- Discuss the performance advantages and disadvantages of micro-surfacing at different RAP contents, and demonstrate the possible maximum allowable RAP content for recycled micro-surfacing in engineering applications.
- Economic analysis of micro-surfacing containing RAP.

This study explores RAP-containing micro-surfacing from three perspectives: mix design, material performance, and economic analysis. It provides a reference for the application prospects of RAP-containing micro-surfacing and lays a foundation for studying the strength formation mechanisms and weak interface issues. These findings may attract the interest of road maintenance professionals.

## 2. Materials

### 2.1. Asphalt Emulsified

**Figure 3:**
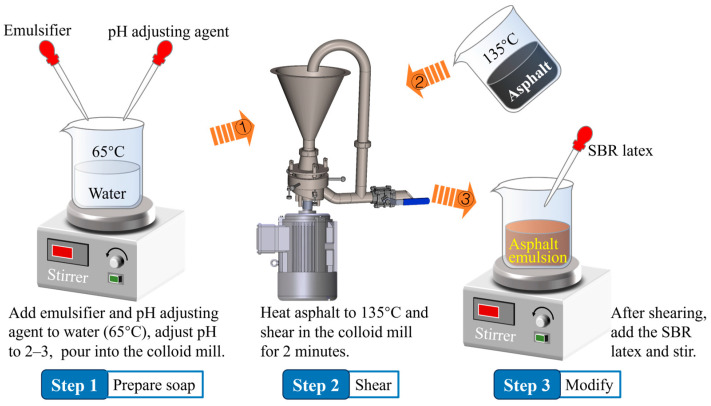
Flow chart of SBR modified asphalt emulsified preparation.

The SBR cationic-modified asphalt emulsion used in this study was laboratory-made, and the production process followed the “post-adding method”, which is the simplest preparation approach and ensures relatively stable emulsion quality [22]. SBR is a commonly used additive in road materials, with a high molecular weight and degree of polymerization, which can improve the low-temperature performance and enhance the high-temperature performance of asphalt. The base asphalt used was 70#, with an SBR modifier content of 3.8%, and it was prepared through colloid mill shearing. The solid content of the emulsion was 63%, and the preparation flowchart is shown in Figure 3. The performance indicators were tested according to ASTM specifications, and the test results are presented in Table 1.

### 2.2. RAP and Aggregates

In this study, the virgin aggregate was basalt, sourced from the highway maintenance base in Wannian County, Jiangxi Province. After sieving through No. 4 and No. 8 sieves, two size ranges of aggregate were obtained: 0–3 mm and 3–5 mm, as show in Table 2. The sand equivalent, crushing value, and other properties of the aggregate were tested according to the standards, and the results met all the requirements.

The RAP aggregate was sourced from the same base, consisting of milled asphalt pavement from highway surface layers, and was crushed into 0–8 mm RAP. The original aggregate was diabase. The particle size distribution was determined through sieving, and the asphalt content was measured to be 4.34%. To ensure consistent moisture content, freshly crushed RAP was selected. Additionally, oversized RAP particles may occur during standard production, which can lead to difficulties in achieving smooth paving during construction. Therefore, strict sieving is necessary to remove oversized particles.

### 2.3. Filler

The micro-surfacing pavement contains a high proportion of fine aggregates. According to ISSA guidelines A–143, MS-3 recommends 0–3% filler [23]. The primary role of filler is to improve the aggregate gradation, fill the voids, and form asphalt mastic with asphalt to enhance the strength and stability of the mixture. Additionally, cement undergoes a hydration reaction, producing calcium silicate hydrate (C–S–H) in a fibrous form, which connects the interface between new and old asphalt and improves the overall strength of the mixture [24]. Due to its large specific surface area and surface acidity or alkalinity, filler can cause premature breaking of the emulsion in slurry mixtures. Adjusting the type and amount of filler can regulate the mixing time of the mixture.

In this study, limestone powder and PO.42.5 cement were selected as fillers. Tests confirmed they met the requirements, and their particle size distribution is shown in Table 2. The cement content was set at 1.5% of the aggregate mass, while the limestone powder content was adjusted as needed and included in the gradation calculation.

## 3. Experimental Methods

The main objective of this study is to investigate the use of RAP as a replacement for virgin aggregate in micro-surfacing. To this end, a mix design process for micro-surfacing containing RAP was proposed based on the ISSA guidelines. Subsequently, six optimal micro-surfacing samples with different RAP contents were prepared according to the standard requirements, and their performance was tested. This included consistency and mixing time tests to determine the optimal water content; Wet-Track Abrasion Loss (WTAL) tests and loaded wheel tracking (LWT) tests to determine the optimal asphalt content, as well as displacement deformation rate; and cohesion tests to validate the mix design. The effects of RAP, asphalt emulsified, and water on the mix design were analyzed.

After successfully meeting all test requirements in this phase, special specimens were prepared to evaluate the surface friction and deformation resistance of micro-surfacing with varying RAP contents, using British pendulum tests and rutting tests. Finally, based on practical construction conditions and cost analysis, the estimated RAP utilization and cost savings were calculated. The maximum allowable RAP content was discussed in combination with pavement performance results.

### 3.1. Mix Design

#### 3.1.1. Estimate Asphalt Emulsion Requirement

The initial asphalt content was determined using the aggregate surface area method, which considers factors such as differences in the surface area and the apparent density of different aggregates. This approach allows for a more accurate calculation of an asphalt content value closer to the optimal amount compared to the empirical method. According to the calculation method provided in ISSA TB-118 [25], the total asphalt content was designed based on an asphalt film thickness of 8 µm. The total asphalt content was then divided by the solid content of the asphalt emulsion, and three initial asphalt emulsion contents were determined by adjusting the value by ±0.75%.

#### 3.1.2. Determining Water Consumption

**Consistency Test**

Consistency is used to determine the appropriate water content in the slurry mixture to ensure workability. It prevents excessive consistency, which could cause premature breaking of the emulsion and hinder proper paving, and insufficient consistency, which could result in slurry overflow. According to ISSA TB-106 [26], the water content is adjusted until a consistency of 2–3 cm is achieved, and the range of water content is recorded.

**Mix Time Test**

The mixing time test is a critical step in the mix design process for micro-surfacing. This test measures the mixing time of a specific combination of materials in the slurry surfacing system. According to the ISSA TB-113 specification [27], the stirring time after adding the asphalt emulsion to the materials must not be less than 120 s. This ensures that the mixture does not break prematurely, which would prevent proper paving. In this study, multiple tests were conducted using different water contents within the range of consistency test results, and combinations with a mixing time greater than 120 s were recorded.

#### 3.1.3. Determining Asphalt Emulsion Requirement

**1 h Wet-Track Abrasion Loss test**

Insufficient asphalt content may result in inadequate inter-aggregate bonding, which can lead to defects such as cracking and raveling. The WTAL test simulates material loss caused by tire wear underwater immersion conditions, thereby determining the minimum asphalt content. According to ISSA TB-100 [28], the WTAL test conducted on samples soaked for 1 h allows a maximum acceptable mass loss of 538 g/m^2^.

**LWT Sand Adhesion Test**

Excessive asphalt content in micro-surfacing pavements may lead to severe asphalt flushing or densification under heavy traffic loads, which can reduce pavement friction. According to ISSA TB-109 [29], the sand adhesion test determines the maximum asphalt content by sprinkling hot sand on the test sample and applying a load. The maximum acceptable adhered sand is 538 g/m^2^.

#### 3.1.4. Mix Design Verification

**Lateral Displacement Specific Gravity Test**

In this study, standard specimens were prepared using the determined material proportions according to ISSA TB-147 [30]. Under a 57.6 kg load, the specimens were subjected to 1000 rolling cycles, with the maximum acceptable percent lateral displacement (PLD) being 5%.

**Wet Cohesion Test**

Slurry mixtures are required to develop sufficient strength within a short period to withstand traffic loads after opening. According to ISSA TB-139 [31], the relationship between the torque of the mixture and time is tested to evaluate its initial setting and curing process, thereby determining the appropriate traffic opening time for micro-surfacing.

### 3.2. Performance Test

#### 3.2.1. 6D Wet-Track Abrasion Loss Test

Pavement damage caused by water-induced deterioration cannot be ignored, and the effect of RAP on the water sensitivity of the mixture remains unknown. Therefore, in this study, a 6D WTAL test was conducted based on Specification ISSA TB-100 [28]. The aggregate loss of mixtures with different RAP contents was compared before and after water immersion to evaluate the moisture susceptibility of each mixture group.

#### 3.2.2. Rutting Test

**Figure 4:**
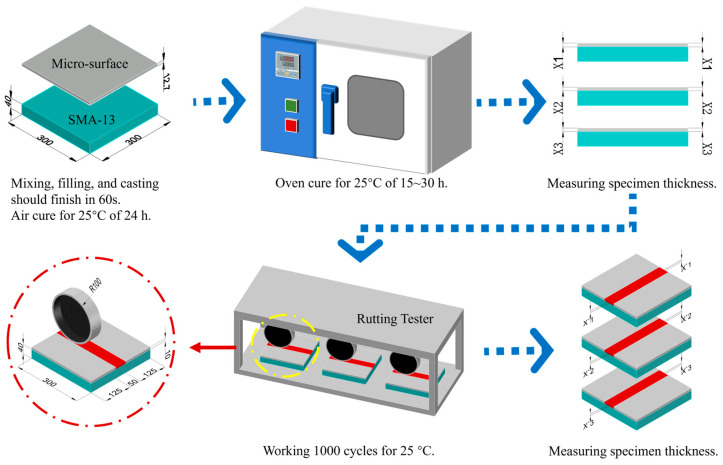
Rutting tester of micro-surface mixture.

To evaluate the rutting resistance of micro-surfacing containing RAP, a rutting test specifically designed for micro-surfacing was conducted, as shown in Figure 4. The bottom layer consisted of a 4 cm SMA-13, which was specially treated to ensure negligible or no rutting. Subsequently, approximately 2400 g of aggregate was used to prepare the slurry mixture based on the determined mix proportions. The mixture was paved and leveled within one minute, with a paving thickness of 1.27 cm. The sample was cured at room temperature for 24 ± 2 h, then placed in a 60 °C oven for 15 to 30 h until constant weight was achieved. After curing, the thickness of the micro-surfacing mixture was measured.

At 25 °C, the specimen was subjected to 1000 passes of loading using a rutting tester, and the rutting depth (*RD*) under load was recorded using a displacement sensor. The percent vertical displacement (PVD) was calculated using Equation (1) to evaluate the effect of RAP content on deformation resistance.

(1) $$PVD=\frac{RD}{X1}\times 100\%$$

where *X*1 = average of vertical measurements before running; *RD* = average of rutting depth.

#### 3.2.3. British Pendulum Test

To evaluate the skid resistance of micro-surfacing containing RAP, the British Pendulum Tester was used to measure the surface skid resistance of each mixture group. Specimens, as shown in Figure 4, were prepared, and the British Pendulum tester Number (BPN) was tested according to ASTM-E303 standards [32].

#### 3.2.4. Economic Analysis

Since replacing virgin aggregates with RAP does not affect construction speed or transportation costs, only material-related costs need to be considered, including emulsified asphalt, virgin aggregates (coarse aggregates, fine aggregates, and fillers), water, and RAP. Considering that replacing virgin aggregates with RAP introduces an additional pre-mixing process, the pre-mixing cost was determined to be USD 0.69/ton after consulting with the construction unit. Therefore, the total material cost (*Cost_mat_*) can be calculated using Equation (2) [33]. The price list for materials and processing costs is shown in Table 3.

(2) $$Cost_{mat}=\sum Material_{i}\;weight\times P_{i}$$

where P*_i_* is the unit price of the materials, USD/Tons.

## 4. Results and Discussion

### 4.1. Mix Design

**Figure 5:**
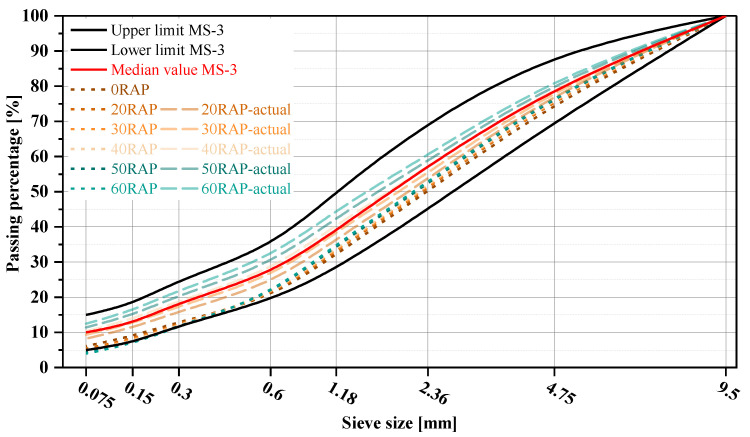
Gradation curve.

Based on the statistical analysis of existing pavement distresses and other data, the MS-3 micro-surfacing mix design was applied with RAP contents of 0%, 20%, 30%, 40%, 50%, and 60%. During the mix design process, RAP was treated as “black rock” and included in the gradation calculations to ensure that the synthetic gradation remained consistent as much as possible. The gradation curves for each group are shown in Figure 5, where the short dash lines represent the synthetic gradation without considering RAP agglomeration, and the dash lines represent the actual gradation after considering RAP agglomeration.

The results indicate that treating RAP as “black rock” during synthetic gradation calculations results in a finer actual gradation below 2.36 mm due to agglomeration. If the synthetic gradation is forced to align closely with the midline of the MS-3 gradation range, the actual gradation below 0.6 mm would exceed the specification limits [34]. Therefore, in this study, aligning the gradation curves closer to the lower limit of the MS-3 gradation range is considered reasonable, ensuring that the actual gradation also falls within the MS-3 specification range.

#### 4.1.1. Estimate Asphalt Emulsion Requirement

The emulsified asphalt content calculated using the surface area method is shown in Table 4. It can be observed that as the proportion of RAP increases, the required amount of emulsified asphalt decreases. This is because higher RAP content results in coarser gradation, and larger aggregate particles have a smaller specific surface area, leading to a lower estimated asphalt demand. The estimated values align with existing research findings, as the aged asphalt in RAP can blend with the new asphalt to form effective asphalt with binding properties, thereby reducing the asphalt demand with the addition of RAP [10].

#### 4.1.2. Determining Water Consumption

**Figure 6:**
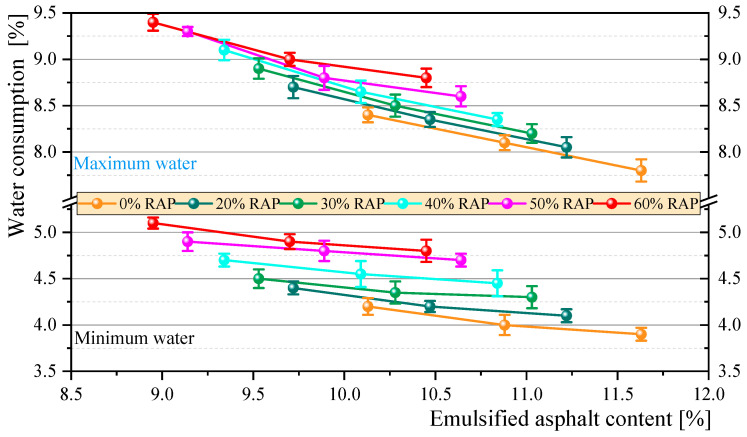
Range of additional water.

The results of the consistency test are shown in Figure 6, with detailed data provided in Appendix A.1. The results indicate that as the RAP content increases, the consistency of the mixture also increases. To ensure adequate flowability, the required additional water also increases. The average water consumption increased, with the range shifting from 4–8.1% to 4.9–9.1%.. This is because the surface of RAP is coated with a large amount of densely distributed mineral powder and fine aggregate, giving it a rougher surface texture compared to virgin aggregate. Additionally, the surface of RAP exhibits hydrophobic properties, which means that, under the same amount of water, some aggregate surfaces in mixtures containing RAP remain insufficiently wetted. This results in poorer lubrication provided by water, leading to lower flowability and, thus, higher consistency.

**Figure 7:**
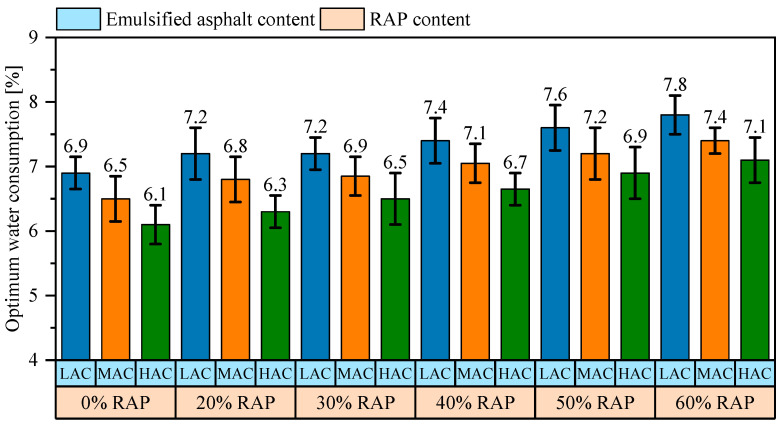
Optimum water consumption. LAC, MAC, and HAC represent low, medium, and high asphalt content, respectively.

The results of the mixing time test are shown in Figure 7, with detailed data provided in Appendix A.2. The results indicate that as the asphalt-aggregate ratio increases, the amount of additional water decreases. This is because the aged asphalt contains anhydride groups, which impart a negative charge to the RAP surface. When RAP comes into contact with emulsified asphalt, the cationic emulsified asphalt preferentially coats the RAP surface. Once emulsified asphalt comes into contact with the aggregate, a portion of the asphalt adheres to the aggregate surface, disrupting the original balance of the emulsified asphalt phase. Subsequently, due to mixing and the adsorption and aggregation among small-sized aggregates, the emulsified asphalt phase becomes imbalanced, leading to the breaking of the slurry mixture. Increasing the emulsified asphalt content allows for more asphalt to remain available after coating the aggregates, thereby delaying the imbalance of the emulsified asphalt phase under external forces. Moreover, emulsified asphalt contains 37% water, which is another primary reason for the reduced additional water requirement.

#### 4.1.3. Determining Asphalt Emulsion Requirement

**Figure 8:**
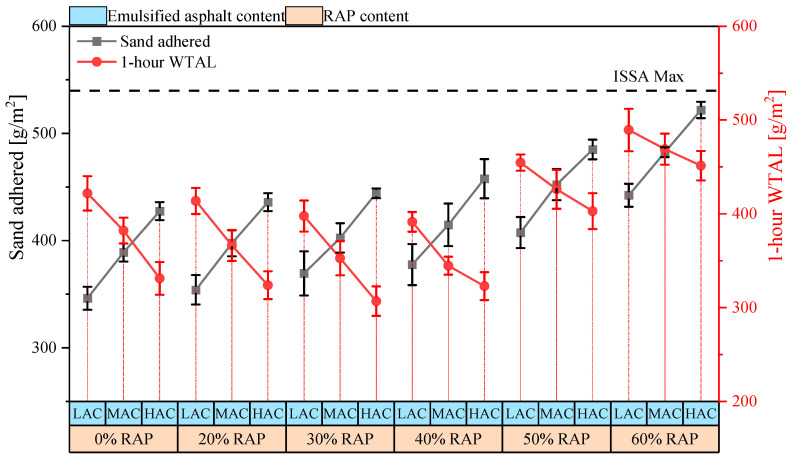
Sand adhesion and abrasion loss test results. LAC, MAC, and HAC represent low, medium, and high asphalt content, respectively.

Figure 8 shows the sand adhesion test results for each mixture, with all samples meeting the specification requirements. However, the sand adhesion values increased with higher RAP content and asphalt-aggregate ratios, which may lead to greater densification of the pavement under heavy traffic, reducing the skid resistance coefficient. The lowest sand adhesion value was observed in the combination without RAP and with an emulsified asphalt content of 10.13%, measuring only 346.2 g/m^2^. In comparison, increasing the emulsified asphalt content by 1.5% resulted in an increase of 81.3 g/m^2^ in sand adhesion, accounting for approximately 23%.

This is because the sand adhesion test determines the sand adhesion value based on the amount of hot sand remaining on the pavement under a specified load. Increasing the emulsified asphalt content inevitably thickens the asphalt film, allowing more hot sand to adhere [35]. Furthermore, for combinations with similar emulsified asphalt content, the sand adhesion value was primarily influenced by the RAP content. Compared to mixtures without RAP, the sand adhesion value increased by approximately 19.7% with 40% RAP content and by about 38% with 60% RAP content. This is mainly because the surface of RAP is already coated with aged asphalt, preventing the new asphalt from being absorbed by the aggregate. Consequently, the asphalt film in mixtures with RAP is thicker than that in mixtures with virgin aggregate, leading to higher sand adhesion values.

In addition, Figure 8 also shows the 1 h WTAL results for each mixture. The results indicate that the abrasion value decreases with an increase in the asphalt-aggregate ratio but initially decreases and then increases as the RAP content rises. At low RAP contents, RAP behaves equivalently to virgin aggregate, and the aged asphalt coating on the RAP surface blends with the new asphalt. Under the same asphalt-aggregate ratio, mixtures containing RAP have a thicker asphalt film, enhancing the adhesion between aggregates and improving abrasion resistance. However, this improvement diminishes when the RAP content exceeds 40%, due to the increased occurrence of agglomeration in RAP. In water immersion and rolling environments, these RAP agglomerates break apart, creating new weak adhesive interfaces because the interior of the RAP agglomerates lacks new asphalt and cement hydration products. This significantly increases the abrasion value.

Using graphical analysis, the optimal asphalt content for each group was determined based on the abrasion value and sand adhesion value for different asphalt contents within the same group. The calculated optimal asphalt contents for RAP contents of 0%, 20%, 30%, 40%, 50%, and 60% are 11.0%, 10.5%, 10.1%, 9.7%, 9.8%, and 9.7%, respectively. It can be observed that RAP reduces the use of emulsified asphalt within a certain limit. However, when the RAP content exceeds 40%, further increases in RAP do not reduce the emulsified asphalt content.

#### 4.1.4. Mix Design Verification

Based on the experimental results, the mix proportions for six different RAP contents are presented in Table 5. The transverse displacement rate and cohesion were also tested to verify whether they meet the required standards.

**Figure 9:**
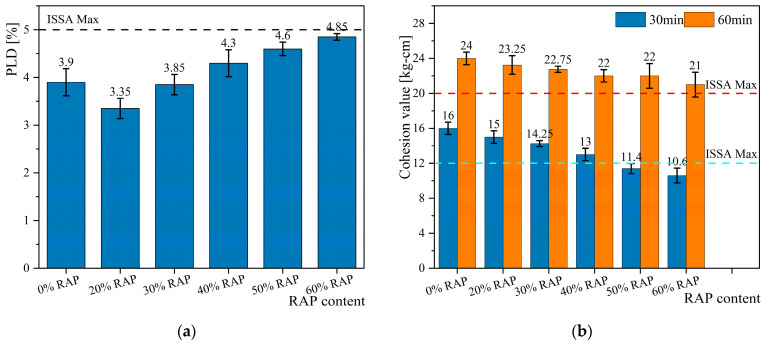
Test results: (**a**) percent lateral displacement; (**b**) cohesion value.

The PLD results, as shown in Figure 9a, indicate that adding 20% RAP can enhance the deformation resistance of the mixture. The PLD of the mixture without RAP is 3.9%, while the PLD with 20% RAP is reduced to 3.35%. This improvement is primarily due to the hydration product of cement, calcium silicate hydrate, which forms fibrous structures. One end of the fibers embeds into the aged asphalt layer on the RAP surface, while the other end is exposed on the asphalt film surface, creating a “reinforcing” effect that enhances the overall strength of the mixture [36,37].

However, when the RAP content exceeds 30%, the PLD increases, reducing the deformation resistance of the mixture. This is likely due to the significant increase in RAP combined with the lower or uneven distribution of cement, resulting in a lack of modulus transition medium between the RAP and new asphalt. Under loading, the weak adhesive interface between the new and aged asphalt fails, leading to an increase in lateral displacement rate.

Additionally, the cohesion results indicate that RAP reduces the early strength of the mixture. As shown in Figure 9b, the 30 min cohesion of the mixture made entirely with virgin aggregates is 16 kg/cm. However, as RAP is added, the cohesion decreases almost linearly, eventually failing to meet the specification requirement of 12 kg/cm. Nevertheless, this does not mean that micro-surfacing containing RAP cannot be applied in engineering projects. The ASTM-D6372 specification mentions that the 30 min cohesion test is primarily used to determine the set time, which indicates when the mixture can no longer be evenly mixed or washed away with water [38]. This implies that mixtures containing RAP require a longer time to release free water, which is caused by the additional water added to meet the required workability time [39,40].

The 60 min cohesion test results similarly show that the inclusion of RAP reduces the strength of the mixture but still meets the requirements for opening to traffic.

The above results demonstrate that the micro-surfacing specimens prepared with the optimal asphalt-aggregate ratio determined by graphical analysis meet the requirements for both displacement rate and cohesion. This indicates that RAP can completely replace virgin aggregates and, within a certain range, can reduce the amount of emulsified asphalt used. However, the required water content will increase by 1–2%, and the formation of early strength will be slower compared to conventional micro-surfacing.

Compared to traditional design methods, this study adjusts the sequence of the mix design tests by first selecting the asphalt content, followed by determining the water content and asphalt content, thereby reducing the number of iterations. This optimized process helps road maintenance workers with less experience to more easily identify the appropriate material composition, rather than adjusting the material proportions through iterative cycles in the design process, thus avoiding an increase in the number of experimental groups.

### 4.2. Performance Evaluation of Micro-Surfacing Containing RAP

#### 4.2.1. Moisture Susceptibility

**Figure 10:**
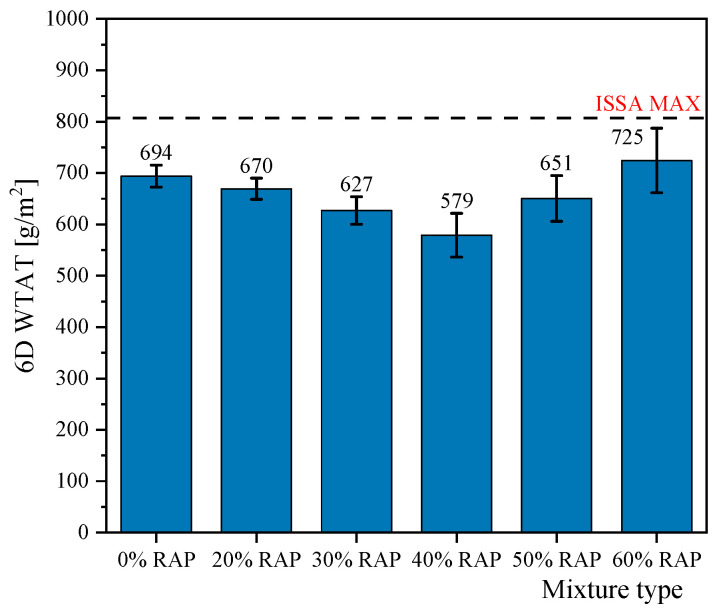
6 Day WTAT test results.

The moisture susceptibility of micro-surfacing specimens decreased after being subjected to 6 days of water immersion at 25 °C, with the best performance observed at 40% RAP content. The results of the 6-day WTAT test are shown in Figure 10. As the RAP content increased, the abrasion loss of the micro-surfacing mixtures initially decreased and then increased, reaching its lowest value of only 579 g/m^2^ at 40% RAP content. This indicates that compared to virgin micro-surfacing mixtures, the inclusion of RAP enhances the stripping resistance. The primary reason is the rough surface of RAP, which allows it to bond more easily with the asphalt mortar, thereby improving adhesion [41]. Additionally, during the curing process after the mixture is paved, the temperature of 60 °C facilitates the slow fusion between the aged asphalt and the new asphalt, transforming RAP from “black rock” into a combination of RAP and asphalt. As a result, recycled mixtures containing RAP have more usable asphalt. It is widely recognized that increasing the asphalt content within a certain range enhances water damage resistance and reduces aggregate stripping [42].

However, when the RAP content exceeds 40%, the stripping loss increases significantly, approaching the ISSA specification limit of 807 g/m^2^. This indicates that RAP is not suitable for excessive use. The most likely reason is that approximately 35% of the RAP exists as weak RAP agglomerates, into which the new binder and SBR modifier cannot penetrate. At low RAP contents, the effect of weak RAP agglomerates breaking under compaction is minimal. However, when the RAP content exceeds 40%, the breakage of agglomerates significantly reduces the overall stripping resistance [43].

#### 4.2.2. Deformation Resistance

**Figure 11:**
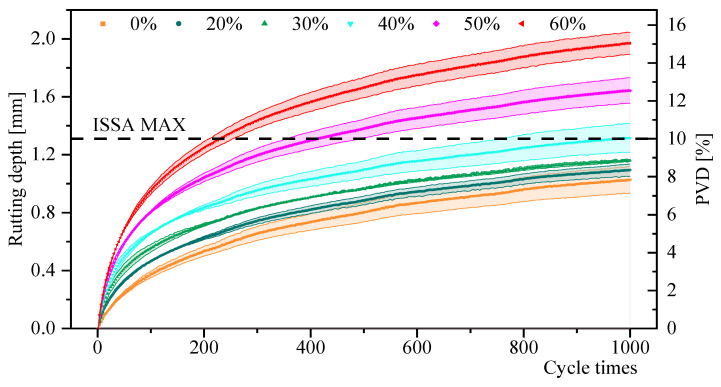
Rutting test results of micro-surface mixture.

**Figure 12:**
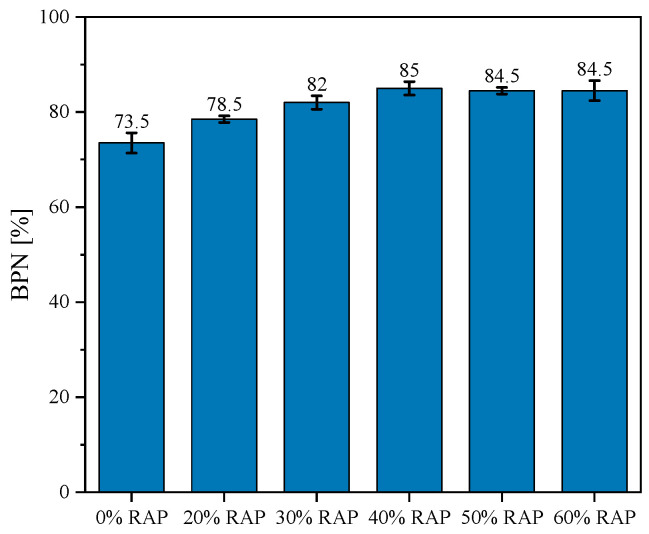
BPN test results.

The rutting test results are shown in Figure 11. Mixtures with low RAP content exhibited better longitudinal deformation resistance. The longitudinal deformation rate of the mixture without RAP was 7.9%, while the deformation rates for mixtures with RAP were 8.3%, 8.9%, 10.0%, 12.5%, and 15.1%, respectively. According to ISSA recommendations, the maximum allowable deformation rate should not exceed 10%, which indicates that the maximum permissible RAP content is 40%. Furthermore, as shown in Figure 12, the rutting depth for RAP contents of 50% and 60% increases significantly higher than the other groups, reaching the maximum deformation rate after 200–400 rolling cycles.

The possible reason for this is that the weak structure of RAP has a significantly higher degree of agglomeration, which negatively impacts the rutting performance of the regenerated mixture [43]. Additionally, micro-surfacing mixtures containing RAP require more water to achieve the desired consistency. As a result, under the same curing conditions, mixtures with RAP tend to have more voids, making them weaker and more susceptible to rutting under load. Further research is needed to investigate these specific causes.

#### 4.2.3. Skid Resistance

The addition of RAP improves the surface skid resistance of micro-surfacing mixtures. The results of the British Pendulum test are shown in Figure 12. The average BPN value of the mixture without RAP was 73.5%, while the average BPN values of mixtures with varying RAP contents were 78.5%, 82%, 85%, 84.5%, and 84.5%, respectively. This indicates that the inclusion of RAP enhances the surface roughness and skid resistance of micro-surfacing pavements. However, this improvement is not evident when the RAP content exceeds 40%.

The primary reason is that the original aggregate in RAP is diabase, which has hardness and angularity properties similar to basalt. Additionally, RAP particles have a richer surface roughness compared to basalt, which increases skid resistance when RAP replaces basalt, similar to how steel slag improves skid resistance when used as a replacement for aggregate [44]. However, high RAP content does not further enhance pavement roughness because the skid resistance of micro-surfacing is influenced not only by the aggregate but also by the asphalt mastic. An increased amount of asphalt mastic reduces skid resistance [45,46].

### 4.3. Economic Analysis

**Figure 13:**
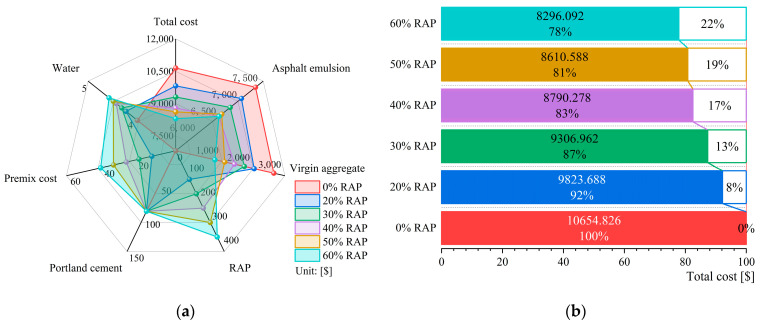
Costing results for materials: (**a**) cost distribution; (**b**) total material cost.

Based on Equation (2) and Table 3, the costs for each group were calculated, taking the paving of 1 km as an example. The cost distribution is shown in Figure 13a. The results indicate that as the RAP content increases, the costs of emulsified asphalt and virgin aggregate decrease significantly, while the costs of RAP and pre-mixing increase slightly due to their lower unit prices. Additionally, the cost of cement remains the same across all groups, and the cost of water is nearly identical.

Figure 13b shows the total material cost calculation results. The highest cost is for the mixture without RAP, with a cost of approximately USD 10,654 per kilometer, while the lowest cost is for the mixture with 60% RAP, with a cost of approximately USD 8296, achieving a cost reduction of about 22%. Furthermore, the combination with 40% RAP content, which has the best overall performance, can also reduce costs by approximately 17%.

It is important to note that the above calculations do not include future maintenance costs. A comprehensive life-cycle cost calculation and environmental assessment require support from actual engineering projects to be determined [47].

## 5. Conclusions

The current micro-surfacing standards lack guidance on the utilization of solid waste. To address this issue, this study aims to explore the application of RAP in SBR-modified asphalt micro-surfacing. We propose a mix design process suitable for micro-surfacing with RAP, investigate the effects of different material compositions on the performance of micro-surfacing, and discuss the optimal RAP content based on both performance and economic factors. The following conclusions are drawn:

1. The proposed micro-surfacing mix design process is suitable for micro-surfacing with RAP and, compared to traditional methods, can reduce the number of test groups required.
2. Replacing virgin aggregates with RAP reduces the demand for SBR-modified emulsified asphalt. When the RAP content is below 40%, each 10% increase in RAP reduces the emulsified asphalt requirement by approximately 0.4%. Beyond 40%, the reduction in emulsified asphalt usage slows down. Additionally, higher RAP content increases water demand, primarily due to differences in the surface characteristics of RAP and aggregates.
3. As the RAP content increases, the surface skid resistance improves, with a maximum increase of 14.9%, mainly due to the rich surface properties of RAP. The water damage resistance first increases and then decreases, with a maximum increase of 16.5%. The influencing factors are complex and require further investigation. Furthermore, the deformation resistance of mixtures containing RAP decreases, particularly when RAP content exceeds 40%, where rutting depth increases significantly. This is due to the breakdown of weak aggregates under wheel loading, leading to structural damage, which is a key limiting factor for RAP content.
4. With the increase in RAP content, the material cost of micro-surfacing decreases, with a maximum reduction of 22%. At the optimal 40% RAP content, material costs can be reduced by 17%, mainly due to the reduced costs of virgin aggregates and emulsified asphalt.

This study proposes an effective micro-surfacing design method based on solid waste utilization and has been validated. However, the focus of this paper is on mix design and performance verification, and further specialized testing can be conducted. Additionally, practical engineering applications and long-term performance studies can be explored.

Currently, most studies on micro-surfacing containing solid waste focus on macro- and meso-scale testing, while smaller-scale explorations, such as the bonding behavior between SBR polymers and RAP surfaces, are insufficient. Future research should focus on the strength formation mechanisms of RAP replacing virgin aggregates and the weak interface behavior in cold recycled micro-surfacing mixtures, including aspects such as RAP aggregation and durability.

## Figures and Tables

**Table 1:** Properties of SBR asphalt emulsion and residual asphalt.

Items		Unit	Results	Specification Requirements	ASTM
Particle charge		/	Positive [+]	Positive [+]	D244
Sieve content		%	0.06	<0.1	D6933
Storage stability	1 d	%	0.5	≤1	D6930
	5 d		4.2	≤5	
Asphalt emulsion residue		%	63	≥62	D6997
Residual asphalt					
Penetration		0.1 mm	58	40~100	D5
Softening point		°C	61.5	≥57	D36
Ductility @ 5 °C		cm	65	≥20	D113

**Table 2:** Test result of the aggregate gradation.

Size [mm]		13.2	9.5	4.75	2.36	1.18	0.6	0.3	0.15	0.075
0–3	Passing rate [%]	100	99.5	69	44	26	16.5	11	7	100
3–5		100	35	15	9.5	6.3	4.2	2.2	1.8	100
Stone powder		100	100	100	100	100	100	100	97.5	95.4
Portland cement		100	100	100	100	100	100	100	99	94
RAP		100	100	79.3	53.8	36.4	20.4	10.0	4.5	1.6

**Table 3:** Cost of materials.

Material	Unit	Value
Virgin aggregate	USD/Ton	28.81
RAP	USD/Ton	5.49
Asphalt emulsion	USD/Ton	617.40
Portland cement	USD/Ton	54.88
Water	USD/Ton	0.56
Premix cost	USD/Ton	0.69

**Table 4:** Asphalt estimate requirement.

Groups	Calculated Value [%]		Preliminary Asphalt Emulsion [%]
	Asphalt-Aggregate Ratio	Asphalt Emulsion	
0% RAP	7.25	10.88	10.13, 10.88, 11.63 ^1^
20% RAP	6.99	10.47	9.72, 10.47, 11.22
30% RAP	6.87	10.28	9.53, 10.28, 11.03
40% RAP	6.75	10.09	9.34, 10.09, 10.84
50% RAP	6.63	9.89	9.14, 9.89, 10.64
60% RAP	6.52	9.70	8.95, 9.70, 10.45

^1^ In subsequent descriptions, for the same group of mixtures, the asphalt content will be named as LAC, MAC, and HAC in order from low to high.

**Table 5:** Mixture composition.

RAP Content [%]	Virgin Aggregate [%]	Polant Cement [%]	Asphalt Emulsion [%]	Water Consumption [%]
0	98.5	1.5	11.0	6.6
20	78.5	1.5	10.5	6.8
30	68.5	1.5	10.1	6.9
40	58.5	1.5	9.7	7.2
50	48.5	1.5	9.8	7.3
60	38.5	1.5	9.7	7.4

## Data Availability

The original contributions presented in this study are included in the article. Further inquiries can be directed to the corresponding author.

## Abbreviations

reclaimed asphalt pavement	RAP	percent lateral displacement	PLD
styrene butadiene rubber	SBR	rutting depth	RD
reclaimed asphalt shingles	RAS	percent vertical displacement	PVD
calcium silicate hydrate	C–S–H	material cost	Cost_mat_
Wet-Track Abrasion Loss	WTAL	British Pendulum tester Number	BPN
Loaded Wheel Tracking	LWT	low asphalt content	LAC
medium asphalt content	MAC	high asphalt content	HAC

## Appendix A

### Appendix A.1

**Table A1:** Range of additional water.

Groups	Asphalt-Aggregate Ratio [%]	Additional Water Consumption [%]	
		Consistency of 2 cm	Consistency of 3 cm
0% RAP	10.13	4.2	8.6
	10.88	4	8.1
	11.63	3.9	7.8
20% RAP	9.72	4.5	8.9
	10.47	4.3	8.4
	11.22	4.1	8.05
30% RAP	9.53	4.5	8.9
	10.28	4.35	8.45
	11.03	4.3	8.2
40% RAP	9.34	4.7	9.15
	10.09	4.55	8.65
	10.84	4.45	8.35
50% RAP	9.14	4.9	9.3
	9.89	4.8	8.8
	10.64	4.7	8.6
60% RAP	8.95	5.1	9.4
	9.70	4.9	9
	10.45	4.8	8.8

### Appendix A.2

**Table A2:** Optimum water consumption.

Groups	Asphalt-Aggregate Ratio [%]	Additional Water Consumption [%]	Mix Time [s]
0% RAP	10.13	6.9	124
	10.88	6.5	127
	11.63	6.1	128
20% RAP	9.72	7.2	125
	10.47	6.8	125
	11.22	6.3	123
30% RAP	9.53	7.2	121
	10.28	6.85	126
	11.03	6.5	125
40% RAP	9.34	7.4	122
	10.09	7.05	124
	10.84	6.65	125
50% RAP	9.14	7.6	120
	9.89	7.2	124
	10.64	6.9	125
60% RAP	8.95	7.8	122
	9.70	7.4	126
	10.45	7.1	126
